# Making nanostructured materials from maize, milk and malacostraca

**DOI:** 10.1038/s41598-021-04001-4

**Published:** 2021-12-24

**Authors:** Subramanian Suriyanarayanan, Ian A. Nicholls

**Affiliations:** Bioorganic and Biophysical Chemistry Laboratory, Linnaeus Centre for Biomaterials Chemistry, Department of Chemistry and Biomedical Sciences, Linnaeus University, 39231 Kalmar, Sweden

**Keywords:** Self-assembly, Nanocomposites

## Abstract

Nano-structured materials are used in electronics, diagnostics, therapeutics, smart packaging, energy management and textiles, areas critical for society and quality of life. However, their fabrication often places high demands on limited natural resources. Accordingly, renewable sources for the feedstocks used in their production are highly desirable. We demonstrate the use of readily available biopolymers derived from maize (zein), milk (casein) and malacostraca (crab-shell derived chitin) in conjunction with sacrificial templates, self-assembled monodisperse latex beads and anodized aluminium membranes, for producing robust surfaces coated with highly regular hyperporous networks or wire-like morphological features, respectively. The utility of this facile strategy for nano-structuring of biopolymers was demonstrated in a surface based-sensing application, where biotin-selective binding sites were generated in the zein-based nano-structured hyperporous network.

## Introduction

The nano- and micro-scale morphologies of materials are critical for their use in many applications where efficient mass-transfer is important, e.g., biomaterials, heterogenous catalysis, separation technology and bio- and chemo-sensing^1–8^. The development of sustainable methods facilitating the large-scale production of materials with pre-determined nano- and micro-scale morphologies and physico-chemical properties is therefore highly desirable^9–12^. Sacrificial templates offer a valuable tool for generating such morphological features, as has been demonstrated using a variety of template materials^13^ ranging from hard porous membranes, e.g., anodized alumina membranes (AAM)^14,15^ and silica beads^16,17^, to soft monodispersed latex (polystyrene) beads (LB)^17–19^ and micelles^20–22^, each of which can be selectively removed after being used to guide the formation of the desired material architecture^23^. Readily available biopolymers^24–27^, e.g. oligopeptides (proteins) and oligosaccharides, are finding increased use in technology-focused applications^28–31^, therefore offering the potential for use in nanomaterial developments due to the diversity of physico-chemical features biomacromolecules can offer in conjunction with the potential for them being obtained from sustainable sources^32,33^. To demonstrate the possibility of using biopolymers for templated nano-material fabrication, studies were first performed using zein^34–38^, a storage protein in maize^39–46^, as a feedstock together with latex beads (LB) and anodized alumina membrane (AAM) as sacrificial templates for generating protein-based long-range nano-structured materials. In this study, we use LB and AAM as sacrificial structures to synthesize protein (zein and casein) and oligosaccharide (chitosan) nano-structured surfaces. For comparative purposes, zein-derived thin films were also prepared in the absence of sacrificial template.

**Figure 1:**
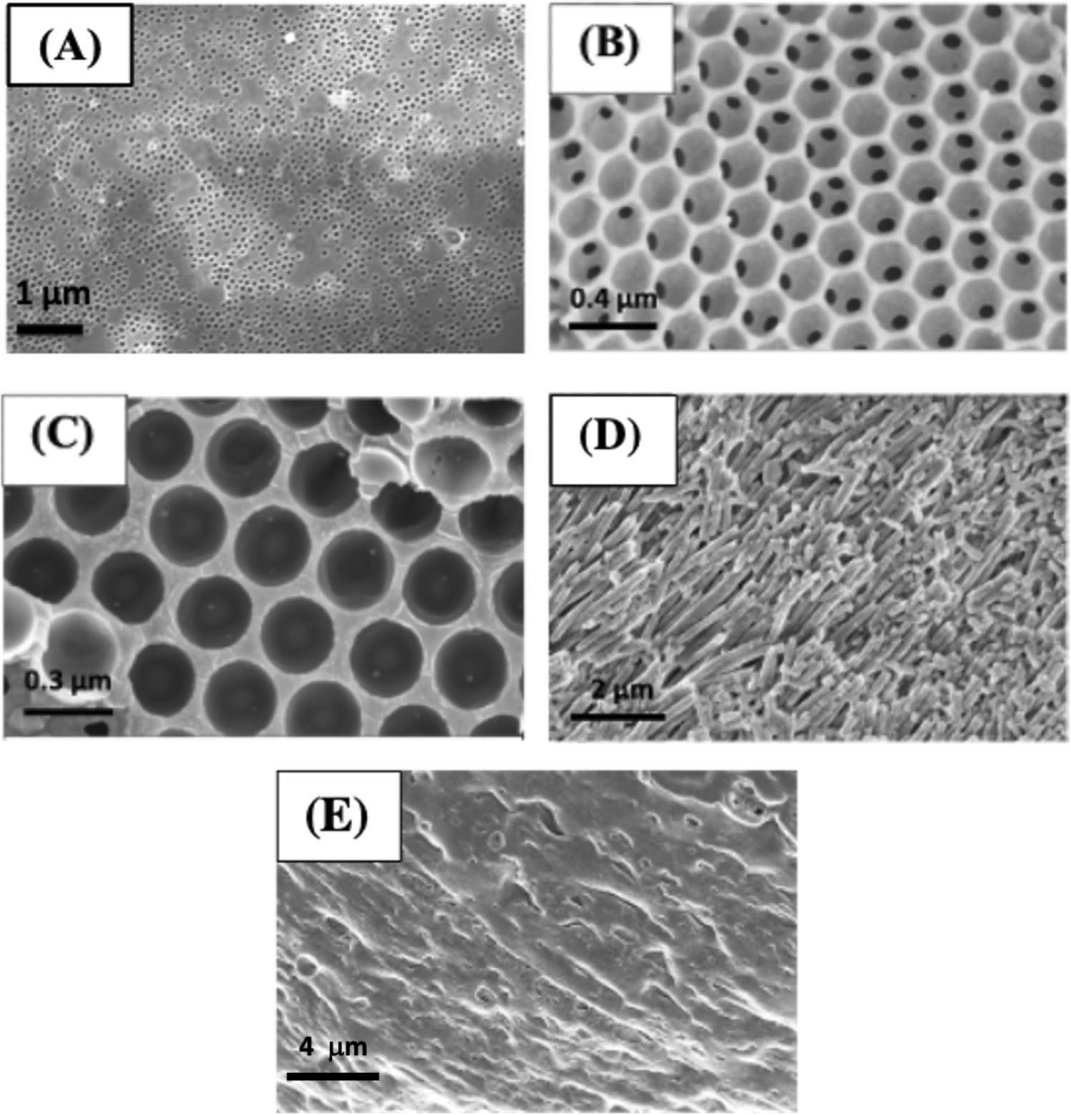
SEM images of (**A**) Z-LB1, (**B**) Z-LB3, (**C**) Z-LB8 and (**D**) Z-AAM (120-nm pore diameter). (**E**) Z-MeOH thin-film prepared from methanol as shown in Table 1-SI.

Sacrificial LB template surfaces were prepared using aqueous solutions of monodispersed latex (polystyrene) beads [100 (LB1), 300 (LB3) or 800 nm (LB8)] which were drop-coated on functionalized Au/quartz or silicon wafer (Scheme 1-SI) and residual solvent (water) evaporated (Sect. 1.3.2 in SI). The morphologies of the latex bead-coated surfaces were examined by scanning electron microscopy (SEM), which revealed highly compact and uniform lattice-like arrangement of the beads with long-range uniformity (see also, Fig. 1A–C-SI). In the case of AAM, with well-defined cylindrical nanopores (Fig. 1D-SI), membranes were placed directly on the substrate surfaces.

**Figure 2:**
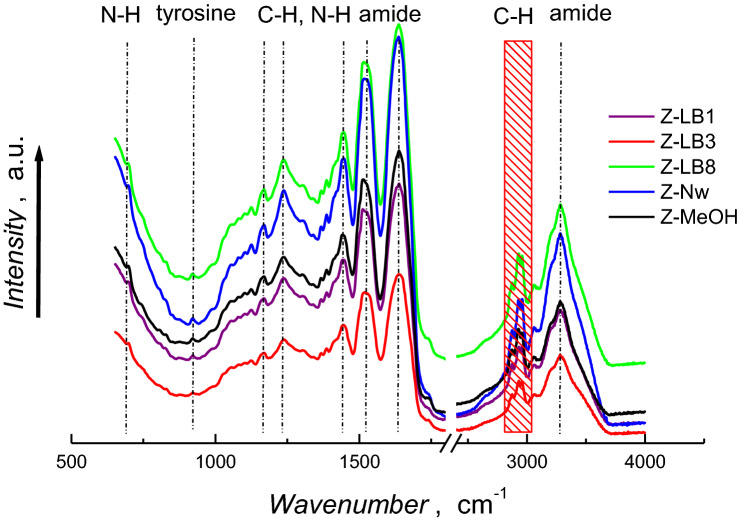
IR spectra of Z-LB1, Z-LB3, Z-LB8, Z-AAM and Z-MeOH zein films.

Nanostructured zein surfaces were prepared by drop-casting methanolic solutions of defatted-zein on functionalized Au/quartz or silicon wafer in the presence of sacrificial latex beads (Scheme 1-SI) or alumina membrane (Scheme 2-SI), or absence of a sacrificial template (Table 1-SI). After solvent evaporation under vacuum, the LBs and AAM sacrificial templates were selectively dissolved in toluene and aqueous HCl, respectively. SEM images of the zein films after extraction of the LB sacrificial templates showed long-range arrays (mm scale) of interconnected uniform spherical cavities with dimensions reflecting the size of the latex bead used (Figs. 1A–C, 2-SI). Interestingly, the magnitudes of the differences in the resonant frequencies corresponding to masses of the zein film prepared with and without LB on Au/quartz surfaces decreased with increased bead size (Table 2-SI). This can be attributed to the larger cavities (lower polymer density) present in the films prepared with the larger beads.

**Figure 3:**
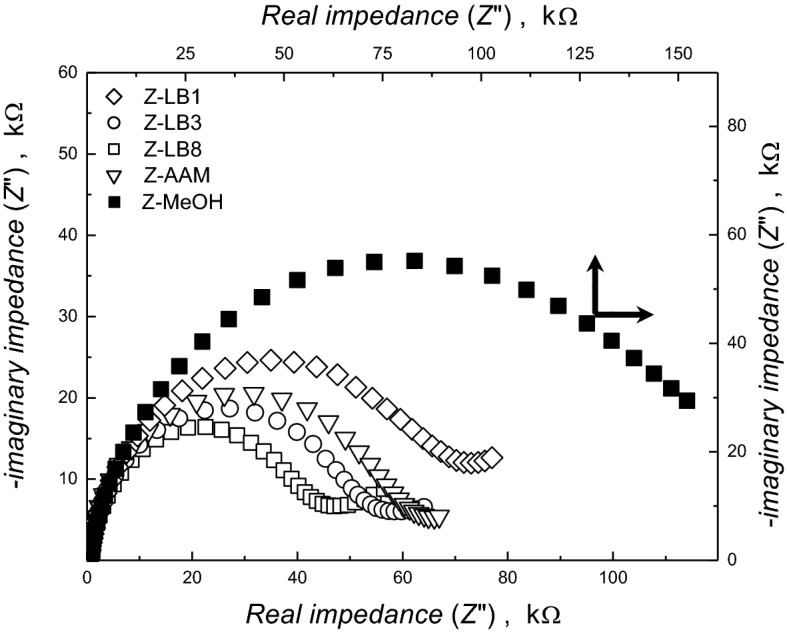
Electrochemical impedance spectra (EIS) showing the complex-plane impedance plot for 2 mM K_4_[Fe(CN)_6_] in KNO_3_ on sacrificial structures templated zein films-coated on Au/quartz electrode. The EIS frequency was scanned from 0.1 Hz to 100 kHz. The impedance curve for Z-MeOH refers to top horizontal and right vertical axis.

In the case of zein films fabricated using AAM as a sacrificial template, SEM images revealed long-range arrays of 150 nm thick coatings of zein-based nanowire-like features (Fig. 1D). The thickness of the nanowires can be manipulated by using AAMs with different pore sizes (Fig. 3B-SI). The lower mass of zein deposited in the presence of AAM (Table 2-SI), relative to the corresponding thin film (Fig. 1E), again demonstrated the lower densities arising from the spacing of the nanowires (Fig. 3A-SI).

Infrared spectra revealed features indicative of defatted zein film (Fig. 2)^47^. Characteristic bands for the predominant alpha-zein conformers corresponding to amine, amide I α-helix, amide I β-sheet, amide II, C–C, amide III stretching, and N–H deformation modes are evident at 3308, 1678, 1623, 1535, 1447, 1238, and 700 cm^−1^, respectively. Importantly, bands at 1678 and 1623 cm^−1^ were not shifted significantly from those of native defatted zein^47^ indicating the conservation of native α-helix and β-sheet structure, respectively. This suggests that the mechanism of interaction between zein proteins in the nanostructured film is similar to that in nature^47–49^. In the LB-templated zein film, characteristic bands for polystyrene (Fig. 4A-SI), δ(C–H) 695, 752, aromatic ν(C–C) 1451, 1492, overtones from 1800 to 2000 cm^−1^ and aromatic ν(C–H) 3023 and 3058 cm^−1^) were absent after extraction with toluene, indicating the efficient removal of the sacrificial LBs.

We then moved on to investigate the impact of the nanostructured zein films on permeability with electrochemical impedance spectroscopy (EIS) using Fe(CN)_6_^3−^/Fe(CN)_6_^4−^ as a redox couple. The real and imaginary components of the complex-plane impedance measure the diffusion of an electroactive redox couple through the porous biopolymer film reflecting. Impedance plot for zein films shows an arc at higher frequencies (Fig. 3) in contrast to the unhindered diffusion-controlled electron transfer reaction on a bare Au surface with a straight line inclined with a gradient of π/4 (Fig. 4B-SI). The diameter of this arc is the measure of the charge transfer resistance (R_ct_) and the diffusion afforded by the biopolymer film for the redox electron transfer reaction. The larger the diameter the greater the resistance to charge transfer or diffusion. The biopolymer film templated with 100 nm beads before extraction with toluene shows higher R_ct_ (135.5 ± 4.3 kΩ) (Table 1, Fig. 3) than the unmodified gold surface (200 Ω) (Fig. 4B-SI). Upon extraction of the templated beads in toluene the R_ct_ value is significantly reduced (68.5 ± 3.1 kΩ) owing to the diffusion of the redox couple via the pathway generated through the interconnected pores in the zein film (Fig. 1A). This behavior was observed for all LB-templated zein films (Table 1). Noticeably, 800 nm LB-templated zein films displayed even lower R_ct_ values (41.7 ± 2.2 kΩ), attributed to the greater pore size enabling more effective diffusion of the redox couple. These decreasing R_ct_ values reflect the more macroporous nature of the zein films obtained when using larger-sized LBs. Thickness of the Z-LB3 film was measured using profilometry and estimated to be 2 µm (Fig. 4C-SI), as compared to the Z-MeOH film (200 nm). Accordingly, the zein film corresponds to 5–6 hyperporous layers of the sacrificial LB3 structures. The number of the hyperporous layers present in a film of similar thickness would be expected to increase with smaller size latex beads.

**Table 1:** Charge transfer resistance offered by the biopolymer coated Au/quartz electrode for Fe(CN)_6_^3−/4−^ redox couple measured using EIS.

Sacrificial template/solvent	Charge transfer resistance *R*_ct_, kΩ* (± S.D.)					
	Zein		Casein		Chitosan	
	Before extraction	After extraction	Before extraction	After extraction	Before extraction	After extraction
LB1	135.5 ± 4.3	68.5 ± 3.1	142.3 ± 3.7	71.2 ± 2.2	142.9 ± 2.1	58 ± 3.2
LB3	132.7 ± 3.1	50.8 ± 2.1	143.4 ± 4.2	48.9 ± 2.2	142.0 ± 3.9	47.9 ± 2.8
LB8	134.4 ± 4.7	41.7 ± 2.2	135.9 ± 3.1	40.2 ± 2.2	144.1 ± 3.7	41.2 ± 1.8
AAM	148.1 ± 5.4	58.9 ± 3.9	-	-	157.6 ± 5.1	52.7 ± 4.1
MeOH/EtOH/AcOH	152.8 ± 2.8	152.2 ± 3.0	150.4 ± 2.5	149.2 ± 2.8	164.1 ± 2.1	164.4 ± 1.7

*Values averaged out for three different measurements.

The zein nanowire-coated surfaces were more permeable than thin-film controls (Fig. 1E), as revealed by reduced R_ct_ values when compared to zein provide access or channel for the diffusion for redox couple, though less permeable than the highly porous LB templated zein films (Table 1). The stabilities of the LB- and AAM-derived zein nanostructures were monitored after storage in PBS (pH 7.4) at 20 °C for 6 months (see Sect. 1.4-SI). No significant changes in the zein nanostructured surfaces were observed by SEM (Fig. 5-SI), highlighting the robust nature of these materials.

The potential for deploying zein nano-structured materials to enhance sensor performance through improved mass-transfer was examined by fabricating a series of biotin imprinted zein nanostructures on Au/quartz resonator surfaces. The performance of these surfaces was studied using a quartz crystal microbalance and flow injection analysis (FIA) and compared with their non-imprinted counterparts and non-nanostructured zein coatings (Fig. 6-SI). Selective biotin recognition was demonstrated, and the LB3-templated imprinted materials induced significant enhancements in sensitivity. The hierarchical imprinted material architectures produced by combining molecular imprinting with the use of sacrificial templates illustrated the potential for these materials in applications requiring efficient mass-transfer. It is important to note that the total binding is enhanced by the larger available surface area, which results from contributions for both the non-specific binding and the increased accessibility to sites selective for biotin.

The scope for nano-structuring other readily available biopolymers was examined by replacing zein with the milk protein casein and the crustacean (e.g. Malacostraca, crab) derived oligosaccharide chitosan. SEM revealed that the nano-structured surfaces prepared using sacrificial LB-template displayed features comparable to those obtained using zein (Figs. 7- and 8-SI). When using AAM as a template, chitosan nanowires were readily obtained (Fig. 8B-SI), though casein nanowires did not survive the low pH used for the extraction of alumina membrane. RAIR spectra of the casein and chitosan nanostructures (Figs. 9-, 10-SI) showed the presence of –NH_2_, CONH_2_ and OH functionalities groups comparable to those of the corresponding non-templated biopolymer films Ca-EtOH and CHI-AcOH. The permeability characteristics of the casein and chitosan nanostructured films reflected those obtained with zein (Table 1). However, the casein and chitosan films were not as stable as those produced with zein as evidenced by the deformation of the macroporous structure (Figs. 11- and 12-SI) after storage for 6 months. These observations were reflected in EIS permeability studies, which showed insignificant differences in R_ct_ value for zein films after storage, whereas casein and chitosan films showed a sharp increase in R_ct_ values after storage indicating the onset of deformation. (Table 3-SI). This is in agreement with the reported stability of the zein films to solvents and high ionic strengths^50^.

Nanostructured biopolymer films can be easily obtained using sacrificial templates and readily available and renewable biopolymer feedstocks. This facile *bench-top* method provides access to material morphologies and associated permeabilities that can be used to advantage in situations requiring efficient mass-transfer, as illustrated here by the introduction of hierarchical features into nanostructured zein films through biotin molecular imprinting and use for enhancing biotin detection using a quartz crystal microbalance. The capacity to tailor nanostructure characteristics through choice of sacrificial template and biopolymer opens for the use of biopolymer-based nanostructured materials in a range of surface-based technologies. Studies are underway to explore the broader application of these materials in surface-based sensors and as catalytic supports.

## Supplementary Information

Supplementary Information.
