# Supplementary material for: Making nanostructured materials from maize, milk and malacostraca
Source: Sci Rep. 2021 Dec 24;11:24420. doi: 10.1038/s41598-021-04001-4 (PMC8709840; doi:10.1038/s41598-021-04001-4)
Supplement: Supplementary file 1 — Supplementary Information. [file 41598_2021_4001_MOESM1_ESM.docx]

**Making Nanostructured Materials from Maize, Milk and Malacostraca**

*Subramanian Suriyanarayanan^a^ and Ian A. Nicholls^a^*^*^

^b.^Bioorganic & Biophysical Chemistry Laboratory, Linnæus University Centre for Biomaterials Chemistry, Linnaeus University, SE-391 82 Kalmar, Sweden

**Table of Contents**

**Experimental section**

- 1. **Chemicals and protocols 3**
  2. **Instrumentation and related protocols**
     1. *Electrochemistry and quartz crystal microbalance (QCM) 3*
     2. *Scanning electron microscope (SEM) 4*
     3. *Infrared spectroscopy* **4**
  3. **Synthesis of biopolymer nanostructures**
     1. *Surface functionalization 5*
     2. *Template synthesis of biopolymer nanostructures* **5**
  4. **Stability of biopolymer films 7**
  5. **References 7**

**Schemes and figures**

Scheme 1-SI 8

Scheme 2-SI 9

Figure 1-SI 10

Figure 2-SI 11

Figure 3-SI 12

Figure 4(A)-SI 13

Figure 4(B)-SI 14

Figure 4(C)-SI 15

Figure 5-SI 16

Figure 6-SI 17

Figure 7-SI 18

Figure 8-SI 19

Figure 9-SI 20

Figure 10-SI 21

Figure 11-SI 22

Figure 12-SI 23

Table 1-SI 24

Table 2-SI 25

Table 3-SI 26

**Materials and methods**

**1.1 Chemicals and protocols**

Zein (from maize Mw = 22 kD), casein (from bovine milk Mw = 24 kD), chitosan (deacetylated Mw = 50 kD), latex beads LB1 (0.1 μm), LB3 (0.3 μm), LB8 (0.8 μm), alumina membrane (AAM), silicon wafer, isopropyl alcohol (HPLC grade), acetic acid (AcOH, glacial), ethanol (HPLC grade), hydrogen peroxide (30%), sulphuric acid, potassium hexacyanoferrate(II), potassium hexacyanoferrate(III), sodium nitrate, hydrochloric acid and toluene were procured from sigma aldrich. Except zein and casein, all the chemicals were used without further purification. Fat content in zein and casein were removed based on the procedure reported elsewhere ^1, 2^. Briefly, a known volume of ethanolic solution of zein was dried in a rotary evaporator. A definite volume of HPLC-grade heptane was then added to it, stirred well, filtered, and dried in a rotary evaporator. This process was repeated at least three times till no fat was detected in the filtrate. Commercial casein was purified by washing with acetate buffer (pH 4.6) for three times and dried in vacuum. Casein was defatted by extraction with diethylether for 2 h followed by drying in a stream of N_2_. Purified casein was stored in vacuum desiccator. Ultrapure grade water (resistivity 18.2 MΩ), collected by purifying distilled water using the Milli-Q gradient water filtration system (Millipore, MA, USA), was used for solution preparation and cleaning substrates.

**1.2. Instrumentation and related protocols**

**1.2.1. Electrochemistry and quartz crystal microbalance studies**

Electrochemical measurements (electrochemical impedance spectroscopy (EIS) and cyclic voltammetry (cv)) were performed using a Reference 600 potentiostat/galvanostat (Gamry Instruments, Warminster, PA, USA). The Gamry framework software provided by the manufacturer was used to control the instrument. Gold coated quartz resonators (Au/quartz), platinum coil and Ag|AgCl electrodes (Gamry Instruments) were used as working, counter and reference electrodes, respectively. The EIS and CV measurements were performed in 0.1 M NaNO_3_ containing 5 mM K_4_[Fe(CN)_6_]/K_3_[Fe(CN)_6_] as redox probe. The EIS experimental data were modeled to Randle’s equivalent circuit, composed of solution resistance (Rs) connected in series with the parallel combination of impedance due to Faradic reaction (R_ct_) and double- layer capacitance (C_dl_). The values presented are derived from measurements from three different samples prepared under identical conditions. The mean values along with the standard deviations have been provided. Standard deviations were computed using Origin 6.1 data analysis software (OriginLab Corporation, MA, USA)

A QCM system (Attana Cell 200 and A100, Attana AB, Stockholm, Sweden) furnished with FIA setup was used for piezoelectric microgravimetric analysis. Attester software (Attana AB, Stockholm, Sweden) procured from the manufacturer was used to configure and control The QCM and FIA condition, respectively. AT-cut quartz resonators (sputter-coated on both the sides with 45 mm diameter and 140 nm thick gold over a 10 nm Ti or Cr underlying layer) of 10-MHz fundamental frequency were procured from Attana AB (Stockholm, Sweden). These substrates were first cleaned and functionalized by a known protocol (see section 2.3) prior to its uses for biopolymer film preparation.

**1.2.2. Scanning electron microscope (SEM)**

Morphological features of the biopolymer films, deposited on silicon substrates (see sections were examined with scanning electron microscopy (SEM) under Leo 1550 Gemini instrument (Zeiss) equipped with a field emission electron gun. A thin layer of conducting pallidum film was sputtered on each sample using an LEICA EM SCD 500 sputtering unit and glued on to alumina stubs with a double side black carbon tape before being inserted in the SEM instrument. Samples were mapped using electron beam generated by applying a voltage of 3 kV to the electron gun at 1 × 10^-5^ mbar vacuum.

**1.2.3. Infrared spectroscopy**

Infrared spectra were recorded with Agilent Cary 630 FT-IR Spectrometer with diamond attenuated total reflectance (ATR) sampling accessory. The Diamond ATR accessory equipped with a type IIa diamond crystal, where the polymer coated substrates is placed to be measured. The instrument was controlled with the Agilent MicroLab FT-IR Software for collecting a background spectrum and a sample spectrum. The samples were recorded within 600-4500 cm^-1^ range with 32 scans and 4 cm^-1^ resolutions.

**1.3 Synthesis of biopolymer nanostructures**

**1.3.1 Surface functionalization**

In this study we prepare biopolymer films by drop casting method in the presence and absence of sacrificial templates on piranha cleaned Au/quartz QCM resonators and silicon wafers. Prior to synthesis, Au coated quartz QCM chips were sonicated for 10 minutes in 500 µL of dry ethanol and subsequently 500 µL dry acetone. Then, the QCM chips were cleaned with piranha solution (H_2_SO_4_:H_2_O_2_ 3:1) for 1 minute (*Caution: "Piranha" solution must be handled with extreme care since it is a hazardous oxidizing agent and reacts violently with most organic materials!*). Next, the QCM chips were thoroughly washed with water, sonicated for 10 minutes in dry acetone and stored in vacuum conditions.

Silicon wafers were cut into 5 × 5 mm square pieces were heated at 80°C in piranha solution (H_2_SO_4_:H_2_O_2_ 7:3) for 1 hr. The acid treated Si wafers were washed with excess amount of water and heated at 80°C in corrosive basic medium (Liquor NH_3_ : H_2_O_2_ : H_2_O, 1:1:6) for 1 hr. Subsequently, these substrates were washed thoroughly with copious amount of water to remove any acid or base impurities and were rinsed in a series of solvents 10 minutes twice in each 500 µL (dry toluene, dry tetrahydrofuran, dry acetone) and were subsequently dried with nitrogen gas. Si wafers were modified with

**1.3.2 Template synthesis of biopolymer nanostructures**

Hyperporous nanostructures of biopolymers were prepared by template directed synthesis using sacrificial monodisperse latex (polystyrene) beads as shown in Scheme 2-SI. Initially, 25 µL of 0.25% aqueous solution of monodisperse polystyrene beads is dispensed on the Si wafer and Au-quartz chips (Scheme 1-SI) and this assembly was kept in fume cupboard (air speed 0.5 m/s) at 22°C for 12 h to remove residual solvent (water). Except for casein, biopolymer solution of zein and chitosan prepared for synthesizing nanowires can be used here as well. Nearly, 5mg of casein was mixed with 1 ml of 1:1 ethanol-water mixed solvent and vortexed for 1 min followed by sonication for 2 mins at 21 °C to disperse the casein protein. Then, the pH of the solution was adjusted 7 by using 5 M NaOH. Again, the mixture was vortexed for 1 min. followed by sonication for 20 mins at 21° C until a clear and homogenous phase is obtained. the About 2 µL of the biopolymer solution prepared above was dispensed on latex beads coated substrates and placed inside the vacuum desicator undisturbed for 6 hours for complete evaporation of the solvent. Sacrificial latex beads were removed by selective dissolution in 1 mL of dry toluene for 2 hrs and repeated three time with fresh toluene solvent.

Biopolymer nanowires were prepared by templated directed synthesis using sacrificial nanoporous alumina (Al_2_O_3_) membrane as shown in Scheme 1-SI. Initially, biopolymer solution was prepared by dissolving 10 mg of zein or 10 mg of chitosan in 75 % methanol and 2.5 % acetic acid, respectively, and vortexed for 30 seconds followed by sonicated for 20 mins at 21 °C. Anopore membrane were cut into 5 X 5 mm size and physically placed on Au/quartz or Si surfaces. An aliquot (2 µL) of the biopolymer solution was dispensed on Si wafer or Au-quartz chips overlaid with Al_2_O_3_ membrane and immediately covered with glass cover slips of 3 mm diameter. This setup was kept undisturbed in fume hood for 6 hours allowing for complete evaporation of the solvent. Later, the alumina membrane was selectively dissolved by immersing the polymer coated substrate in 1 mL of 1.2 M HCl at 35°C for 12 hrs.

Nanoparticle of zein and casein was prepared by the known procedure reported elsewhere (^3^). About 2.0 grams of casein was added to 100 mL of deionized water and the mixture is stirred well for 12 h at 22°C allowing for full hydration of the protein. Then the pH of the casein solution was regulated to 2.0 with the addition of 6 M HCl and the mixture is stirred for 12 hours at 75° C. The mixture was then purified by washing with deionized water for five times followed by centrifugation at low speed (1000 rpm). Pellet collected casein nanoparticles were freeze-dried for 12 h and then stored in a refrigerator prior its use.

**1.4 Stability measurements**

Stability of biopolymer film were examined using EIS at definite intervals with respect to the permeation characteristics of the nanostructures for electroactive redox couple, after storage in buffered solutions (at pH value close to their pI values), for 24 h, 7 days, 30 days, 6 months and 1 year. Films of zein, casein, and chitosan were stored in PBS (pH 6.5), citrate buffer (pH 4.6), and PBS (pH 6.0), respectively, at 8°C followed by rinsing in deionized water prior to the measurement. Topography of the biopolymer films were also examined by SEM to correlate the stability and morphological features developed under prolonged storage conditions.

**1.5 References**

1. F. W. Douglas, J. Tobias, M. L. Groves, H. M. Farrell and L. F. Edmondson, *Journal of Dairy Science*, 1982, **65**, 339.

2. S. Subramanian and S. Sampath, *Biomacromolecules*, 2007, **8**, 2120.

3. Y. Sun and S. Zhong, *Analytical and Bioanalytical Chemistry*, 2018, **410**, 3133.

4. M. C. Regier, J. D. Taylor, T. Borcyk, Y. Yang and A. K. Pannier, *Journal of Nanobiotechnology*, 2012, **10**, 44.


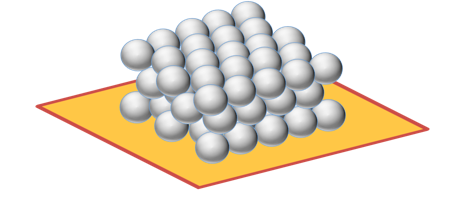

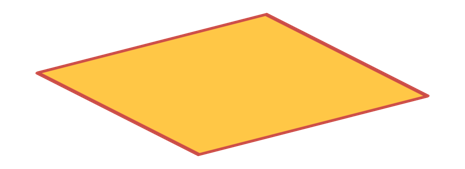

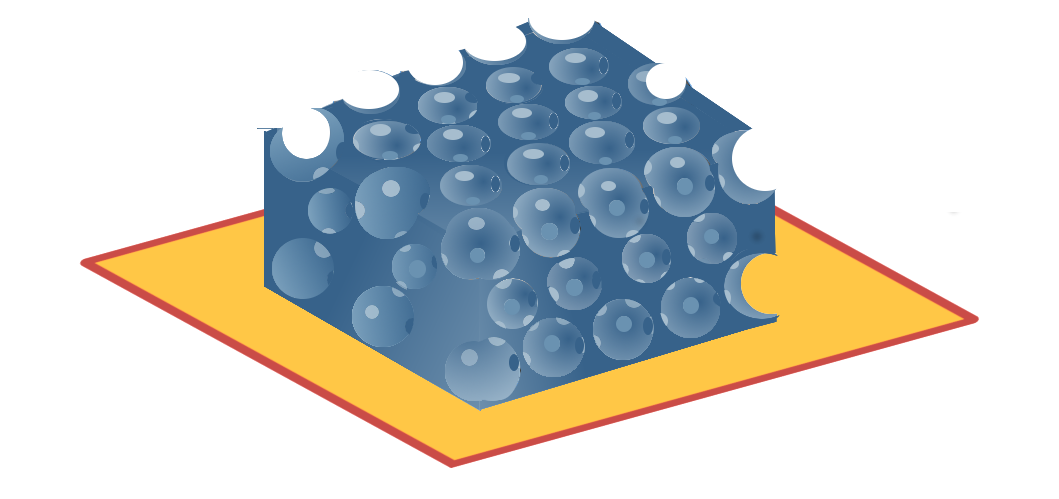

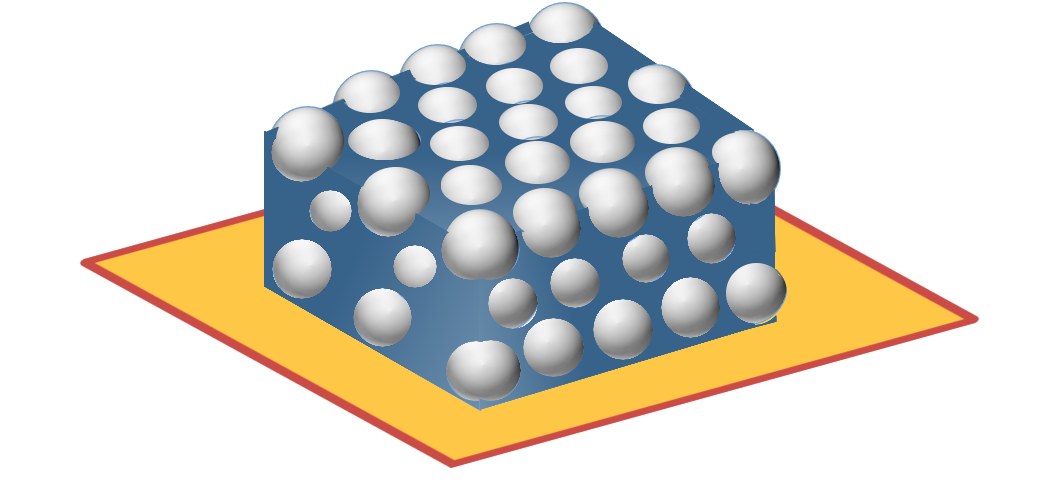

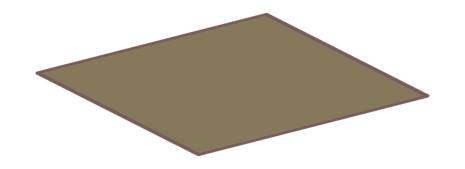


Drop coating of

latex beads

Biopolymer

solution

Solvent

evaporation

Extraction of

latex beads


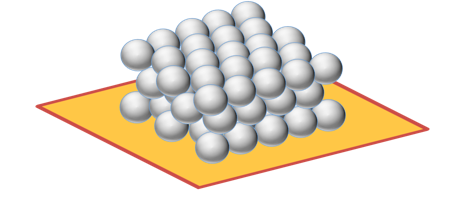

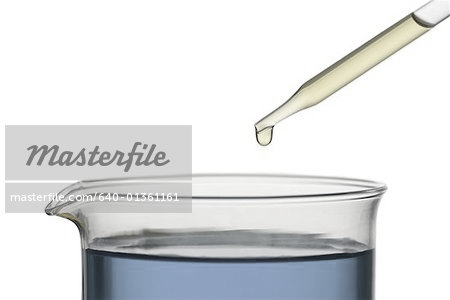

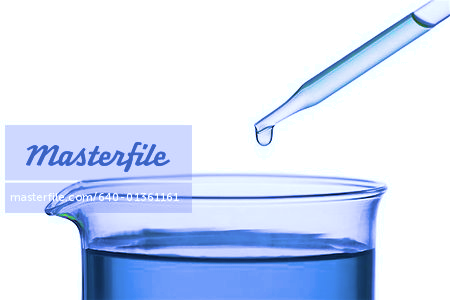


Piranha

cleaning

Si/SiO_2_ or Au

**Scheme 1-SI** Schematic representation for the synthesis of hyper-porous biopolymers from zein, casein and chitosan.


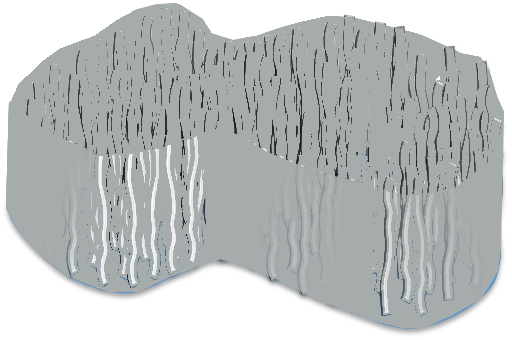


Alumina

membrane


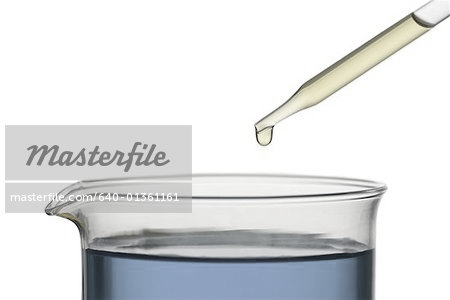

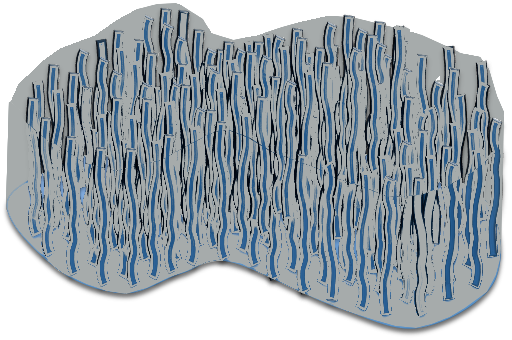

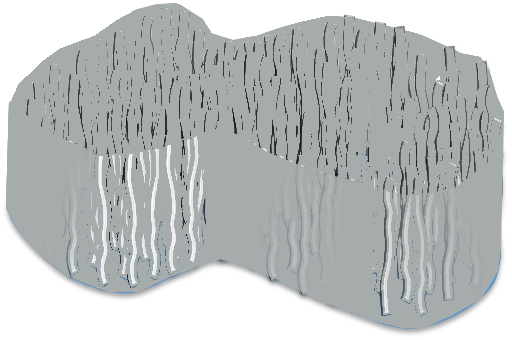


Piranha

cleaning

Biopolymer

solution

Solvent

evaporation

Extraction of

alumina membrane

Si/SiO_2_or Au

**Scheme 2-SI** Schema for the fabrication of biopolymer nanowires from zein and chitosan.

**Figure 1-SI**. SEM images of sacrificial scaffolds (A) LB1, (B) LB3, (C) LB8 and (D) alumina membrane self-assembled on Si wafer

Figure 2-SI Topography of zein film templated LB3 mapped with SEM at (A) 73, (B) 1.7, (C) 2.6 and (D) 4.5 kX magnification scale.

Figure 3-SI Topography of zein film prepared in the presence of (A) 120 nm and (B) 40-nm pore diametered-AAM by drop casting from methanolic solution.

(i)

(ii)


(iii)

Figure 4(A)-SI. IR spectra of (i) Z-LB1, (ii) Z-LB3 and (iii) Z-LB8 film before and after extraction of latex beads in toluene. Bands marked in the spectra corresponds to δ(C-H) 695, 752, aromatic ν(C-C) 1451, 1492, and aromatic ν(C-H) 3023 and 3058 cm^-1^

Figure 4(B)-SI. Figure 3. Electrochemical impedance spectra (EIS) showing the complex-plane impedance plot for 2 mM K_4_[Fe(CN)_6_] in KNO_3_ on the sacrificial template extracted zein films and Au/quartz electrode. The EIS frequency was scanned from 0.1 Hz to 100 kHz. The impedance curve for the bare gold refers to top horizontal and right vertical axis.

Figure 4(C)-SI. Surface profile of the Z-LB3 film measured using profilometer. Inset is the picture of the zein film on Au/quartz surface. The black line in the inset indicates the path travelled by the profilometer probe.


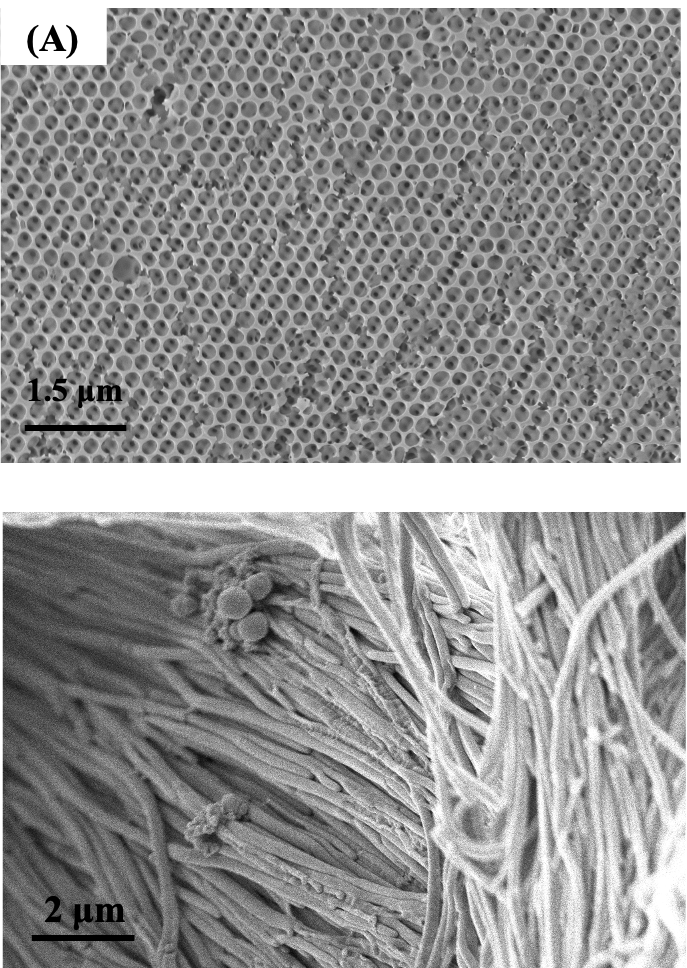


Figure 5-SI. Topography of (A) Z-LB3 and (B) Z-AAM observed through SEM after prolonged storage in buffered solution for 6 months (see section 1.5 in SI)

Figure. 6-SI. Resonant frequency change *vs.* concentration of biotinmethylester (BtOMe) for the repeated injection of BtOMe under chosen FIA condition, on the Au/quartz resonator coated with biotin-imprinted zein film (BMZ-LB3 and BMZ-MeOH) and non-imprinted zein films (RZ-LB3 and Rz-MeOH). FIA conditions: Carrier buffer: phosphate buffer (0.01 M) containing 150 mM NaCl at pH = 6.5. flow rate and volume of the injected analyte was adjusted to 25µL/min and 75 µL, respectively.


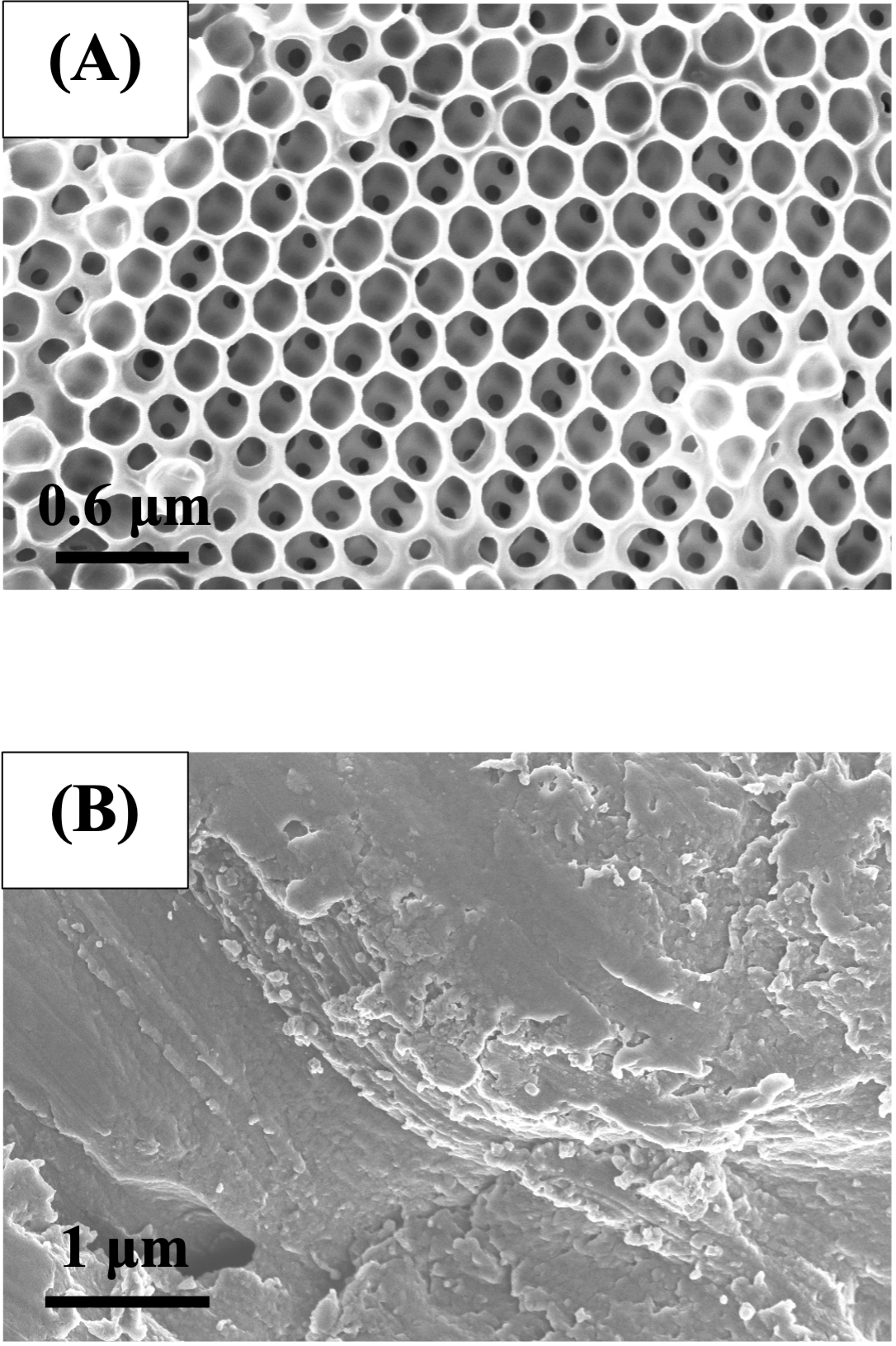


Figure 7-SI. SEM images of (A) casein film templated with LB3 and (B) casein thin film, drop casted on silicon wafers, respectively.

Figure 8-SI. SEM images of chitosan film templated with (A) LB3 as well as (B) AAM and. (C) is the thin film (AcOH/waer), drop casted on silicon wafers, respectively.

Amide I, II and III

δ(C-O)

ν(O-H)

ν(CH_2_)

Figure 9-SI. IR spectra of casein film prepared under different conditions as given in Table 1-SI: (a) Ca-LB1, (b) Ca-LB3, (c) Ca-LB8 and (d) Ca-EtOH.

ν(O-H)

ν(CH_2_)

ν(C=O)

ν(N-H)

δ(C-O) δ(O-H)

Figure 10-SI. IR spectra of chitosan film prepared under different conditions as given in Table 1-SI: (a) CHI-LB1, (b) CHI-LB3, (c) CHI-LB8, (d) CHI-AAM and (e) CHI-AcOH.


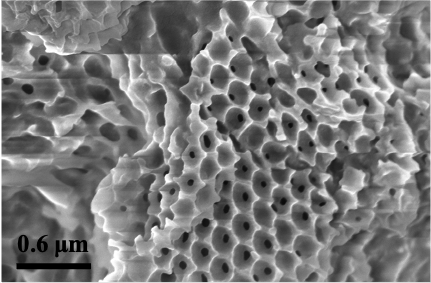


Figure 11-SI. Topography of casein templated with LB3 observed through SEM after prolonged storage in buffered solution for 6 months (see section 1.5 in SI)


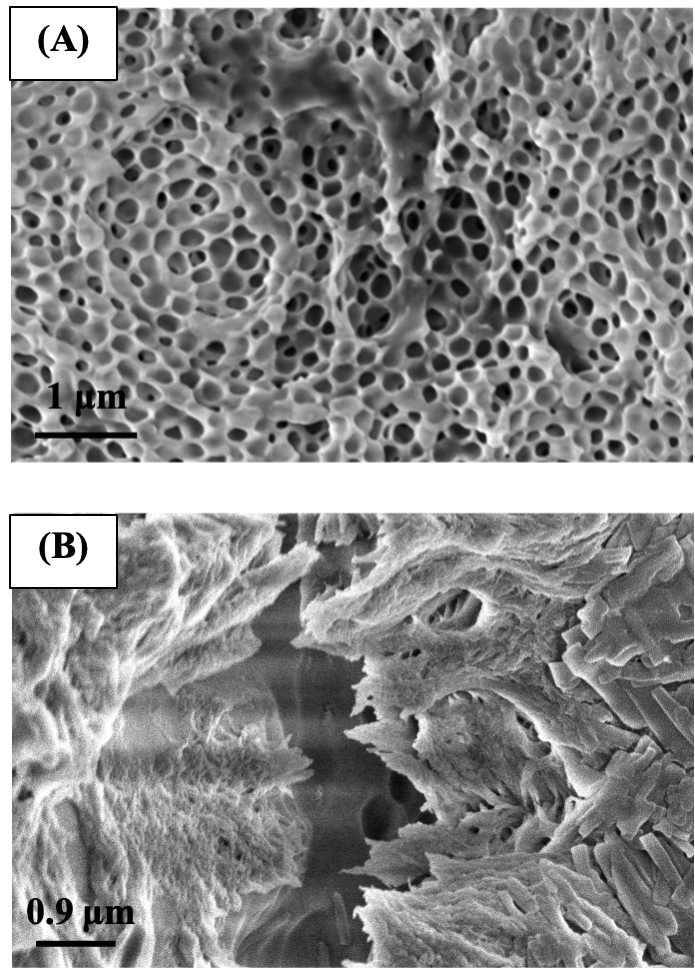


Figure 12-SI. Topography of chitosan film templated with (A) LB3 and (B) AAM observed through SEM after prolonged storage in buffered solution for 6 months (see section 1.5 in SI)

Table 1-SI. List of biopolymer films prepared and characterized in this study.

| System | Biopolymer | Sacrificial template | Solvent | Extraction medium |
| --- | --- | --- | --- | --- |
| Z-LB1 | Zein | 0.1 μm-latex beads | MeOH | Toluene |
| Z-LB3 | Zein | 0.3 μm-latex beads | MeOH | Toluene |
| Z-LB8 | Zein | 0.8 μm-latex beads | MeOH | Toluene |
| Z-AAM | Zein | Alumina membrane | MeOH | HCl (1.2 M) |
| Z-MeOH | Zein | - | MeOH | Water |
| Ca-LB1 | Casein | 0.1 μm-latex beads | EtOH/water | Toluene |
| Ca-LB3 | Casein | 0.3 μm-latex beads | EtOH/water | Toluene |
| Ca-LB8 | Casein | 0.8 μm-latex beads | EtOH/water | Toluene |
| Ca-EtOH | Casein | - | EtOHWater | Water |
| CHI-LB1 | Chitosan | 0.1 μm-latex beads | AcOH/water | Toluene |
| CHI-LB3 | Chitosan | 0.3 μm-latex beads | AcOH/water | Toluene |
| CHI-LB8 | Chitosan | 0.8 μm-latex beads | AcOH/water | Toluene |
| CHI-AAM | Chitosan | Alumina membrane | AcOH/water | HCl (1.2 M) |
| CHI-AcOH | Chitosan | - | AcOH/water | Water |

| Substrate | Mass of the biopolymer deposited, μg* | | |
| --- | --- | --- | --- |
|  | Zein | Casein | Chitosan |
| Au/quartz/100 nm-latex beads | 2.47 | 2.84 | 3.12 |
| Au/quartz300 nm-latex beads | 0.77 | 0.81 | 1.03 |
| Au/quartz/800 nm-latex beads | 0.45 | 0.49 | 0.59 |
| Au/quartz/alumina membrane | 0.89 |  | 1.11 |
| Au/quartz/film | 3.81 | 4.03 | 4.77 |

Table 2-SI. Piezoelectric microgravimetric (QCM) estimation for the amount of zein deposited prepared under various conditions.

* values averaged out for three different measurements

Table 3-SI.

| Substrate | Charge transfer resistance *R*_ct_, kΩ* (± S.D.) | | | | |
| --- | --- | --- | --- | --- | --- |
|  | 1 day | 7 days | 30 days | 6 months | 1 year |
| Ca-LB3 | 48.9 ± 2.2 | 50.1 ± 2.7 | 49.5 ± 3.3 | 65.2 ± 5.9 | 90.8 ± 5.1 |
| Ca-EtOH | 149.2 ± 2.8 | 152.3 ± 3.2 | 154.6 ± 2.8 | 157.4 ± 4.4 | 156.8 ± 4.5 |
| Z-LB3 | 50.8 ± 2.1 | 52.1 ± 3.2 | 51.5 ± 3.5 | 52.8 ± 3.2 | 55.6 ± 3.5 |
| Z-AAM | 58.9 ± 3.9 | 60.2 ± 4.2 | 60.8 ± 3.2 | 64.5 ± 3.9 | 66.1 ± 3.1 |
| Z-MeOH | 152.2 ± 3.0 | 151.4 ± 5.7 | 151.2 ± 4.2 | 153.7 ± 3.6 | 155.3 ± 3.9 |
| CHI-LB3 | 47.9 ± 2.8 | 49.2 ± 2.9 | 53.5 ± 3.0 | 69.8 ± 2.8 | 86.4 ± 3.3 |
| CHI-AAM | 52.7 ± 4.1 | 52.5 ± 5.5 | 56.6 ± 4.7 | 78.2 ± 6.9 | 108.1 ± 5.9 |
| CHI-AcOH | 164.4 ± 1.7 | 164.9 ± 2.9 | 169.5 ± 2.2 | 183.7 ± 2.9 | 209.2 ±7.2 |

* values averaged out for three different measurements
